# Role of lymph node yield and lymph node ratio in predicting outcomes in non‐metastatic colorectal cancer

**DOI:** 10.1002/bjs5.96

**Published:** 2018-08-08

**Authors:** C. H. A. Lee, S. Wilkins, K. Oliva, M. P. Staples, P. J. McMurrick

**Affiliations:** ^1^ Cabrini Monash University Department of Surgery Cabrini Hospital Malvern Victoria Australia; ^2^ Cabrini Institute Cabrini Hospital Malvern Victoria Australia; ^3^ Department of Epidemiology and Preventive Medicine, School of Public Health and Preventive Medicine Monash University Melbourne Victoria Australia

## Abstract

**Background:**

Lymph node yield (LNY) of 12 or more in resection of colorectal cancer is recommended in current international guidelines. Although a low LNY (less than 12) is associated with poorer outcome in some studies, its prognostic value is unclear in patients with early‐stage colorectal or rectal cancer with a complete pathological response following neoadjuvant therapy. Lymph node ratio (LNR), which reflects the proportion of positive to total nodes obtained, may be more accurate in predicting outcome in stage III colorectal cancer. This study aimed to identify factors correlating with LNY and evaluate the prognostic role of LNY and LNR in colorectal cancer.

**Methods:**

An observational study was performed on patients with colorectal cancer treated at three hospitals in Melbourne, Australia, from January 2010 to March 2016. Association of LNY and LNR with clinical variables was analysed using linear regression. Disease‐free (DFS) and overall (OS) survival were investigated with Cox regression and Kaplan–Meier survival analyses.

**Results:**

Some 1585 resections were analysed. Median follow‐up was 27·1 (range 0·1–71) months. Median LNY was 16 (range 0–86), and was lower for rectal cancers, decreased with increasing age, and increased with increasing stage. High LNY (12 or more) was associated with better DFS in colorectal cancer. Subgroup analysis indicated that low LNY was associated with poorer DFS and OS in stage III colonic cancer, but had no effect on DFS and OS in rectal cancer (stages I–III). Higher LNR was predictive of poorer DFS and OS.

**Conclusion:**

Low LNY (less than 12) was predictive of poor DFS in stage III colonic cancer, but was not a factor for stage I or II colonic disease or any rectal cancer. LNR was a predictive factor in DFS and OS in stage III colonic cancer, but influenced DFS only in rectal cancer.

## Introduction

Oncological resection of colorectal cancer involves removing the tumour with clear margins and harvesting of draining lymph nodes. Adequate lymph node harvest allows accurate staging and minimizes the risk of understaging nodal positive disease. The AJCC/UICC recommend a minimum of 12 lymph nodes should be identified in colorectal cancer specimens^1^, ^2^, ^3^, ^4^. Although lymph node yield (LNY) has been used as a surrogate indicator of adequate surgical resection, a substantial proportion of resections (30–50 per cent) still fall into the low LNY category based on population studies^5^, ^6^, ^7^.

Low LNY has been associated with poorer survival outcome in stage II and III colorectal cancer^7^, ^8^, ^9^, ^10^. A systematic review^11^ concluded that increased LNY was associated with improved survival in stage II–III colonic cancer. LNY was also shown in a large population study to be an independent prognostic factor in node‐negative colorectal cancer irrespective of T category^12^. This study^12^ concluded that stage migration alone contributed to the observed survival difference. Despite a rise in median LNY over recent years, the percentage of stage III colorectal cancers has not increased proportionately^9^,^13^, and at 20 years the Surveillance, Epidemiology, and End Results (SEER) data showed no correlation between the proportion of positive lymph nodes and LNY despite an increase in LNY, suggesting that stage migration alone may not account for improved survival in patients with colorectal cancer^14^.

The prognostic significance of low LNY (less than 12) is debatable in locally advanced rectal cancer treated with neoadjuvant therapy, as LNY is known to be reduced in this setting^14^, ^15^, ^16^, ^17^, ^18^, ^19^. Several studies^20^, ^21^, ^22^ have found no survival difference between low and high LNY groups in this setting. Some studies^16^,^23^, ^24^, ^25^ have suggested that lymph node ratio (LNR) should be used for prognostication rather than LNY in stage III colorectal cancer, as it is more representative of tumour burden.

This study aimed to examine the correlation between clinical variables and LNY, and to examine the prognostic significance of LNY and LNR in stage 0 (complete pathological response, pCR) and stage I–III colorectal cancer.

## Methods

An observational study was performed using the Cabrini Monash University colorectal neoplasia database^26^ of consecutive patients treated for stage I–III colorectal adenocarcinoma under the care of 11 colorectal surgeons at Cabrini, Avenue and Alfred hospitals (Melbourne, Victoria, Australia) between January 2010 and March 2016. These three hospitals treat a mixture of private and public patients, and are broadly representative of all socioeconomic groups within the city of Melbourne. Ethical approval for this study was obtained from Cabrini Human Research Ethics Committee (reference 04‐21‐03‐16).

Oncological resection involved *en bloc* resection of tumour with clear margins and high ligation of vascular pedicles, which ensured adequate lymphadenectomy. Neoadjuvant therapy, either long‐course chemoradiotherapy (nCRT) or short‐course radiotherapy, was routinely offered to patients with locally advanced rectal cancer (T3–4 or node‐positive disease). Patients with pCR after neoadjuvant therapy were included in the study. pCR was defined by the absence of residual tumour cells in the surgical specimen, as described previously^27^. Patients who presented with distant metastasis and/or synchronous colorectal cancer were excluded.

Management decisions were based on multidisciplinary team meetings held before surgery. Resected specimens were examined by the pathology department at the hospital in which the resection took place (the small number of specimens from Avenue Hospital were outsourced to a pathology service). Tumours were staged according to AJCC guidelines (7th edition)^1^. Pathological examination of lymph nodes in resected specimens relied on manual dissection by the pathologists. Low LNY was defined as fewer than 12 nodes in the resected specimen. If the initial LNY was less than 12, a fat clearance technique using Carnoy's solution was performed routinely in two of the three sites to increase the yield. LNR was defined as the ratio of metastatic lymph nodes to the total number of lymph nodes examined. LNR was classified into four tiers based on rectal cancer data published by Danish Colorectal Cancer Group^5^: LNR 1, less than 0·08; LNR 2, 0·08 to less than 0·25; LNR 3, 0·25 to less than 0·50; LNR 4, 0·5–1·0.

All patients had routine follow‐up every 3–6 months for the first 2 years with serial measurement of carcinoembryonic antigen. CT of the chest, abdomen and pelvis was performed annually in addition to colonoscopy as deemed clinically appropriate. After 2 years, follow‐up was usually annual, with CT, estimation of blood tumour markers and colonoscopy for metachronous disease performed at the discretion of the treating surgeon and in line with national guidelines. In data analyses, follow‐up was defined as the time from the date of primary surgery to a patient event, such as disease recurrence or death. There was no minimum duration of follow‐up. Follow‐up information was derived from the colorectal neoplasia database and patient hospital records. After surgery for the primary tumour, patients who developed local recurrence or metastasis were no longer considered disease‐free for statistical analysis. Patients who died at any time, for any reason, after surgery were counted as deaths in overall survival analysis.

### Statistical analysis

Data analysis was performed using Stata^®^ 14 (StataCorp, College Station, Texas, USA). Linear regression (ordinary least squares) was used to analyse the association of LNY and LNR with clinical variables (histological grade, overall stage, tumour site and use of neoadjuvant therapy if rectal cancer). The effects of LNY and LNR on disease‐free (DFS) and overall (OS) survival were investigated using survival analysis techniques (Cox regression, Kaplan–Meier survival analysis and log rank tests). Independent prognostic factors were identified in both univariable and multivariable analyses (Cox regression). χ^2^ tests were used in additional analyses between groups. The significance level was set at 5 per cent, and terms were included in the models when the *P* value was below this level. Robust standard error estimates were obtained to account for the lack of independence between observations for patients with more than one procedure. *P* < 0·050 (two‐tailed) was considered statistically significant.

## Results

A total of 1573 patients fulfilled the selection criteria. Some 1585 resections were performed with curative intent (colonic cancer, 1015 (64·0 per cent); rectal cancer, 570 (36·0 per cent), and 12 were excluded (local recurrence, 2; metachronous colorectal cancer, 10). Of the 1585 resections, 1163 (73·4 per cent) were treated at Cabrini Hospital, 401 (25·3 per cent) at Alfred Hospital and 21 (1·3 per cent) at Avenue Hospital. The median duration of follow‐up was 27·1 (range 0·1–71) months. The median LNY was 16 (range 0–86), and 372 (23·5 per cent) of all resections had an LNY below 12. Median age was 71 (range 22–100) years. Of the 570 patients with rectal cancer, 241 (42·3 per cent) received neoadjuvant therapy; 211 (87·6 per cent) received long‐course nCRT and 30 (12·4 per cent) had short‐course radiotherapy. Patient characteristics and clinicopathological features are summarized in *Table* 1.

**Table 1:** Clinicopathological characteristics and treatment events of patients

	No. of treatment episodes* (*n* = 1585)
Lymph node yield†	17·1(8·2)
Age (years)‡	71·6 (22–100)
Sex ratio (M : F)	809 : 776
ASA grade (*n* = 1584)	
I	329 (20·8)
II	670 (42·3)
III	522 (33·0)
IV	63 (4·0)
Tumour location	
Colon	1015 (64·0)
Rectum	570 (36·0)
Tumour site in colon	
Caecum	167 (10·5)
Ascending colon	208 (13·1)
Hepatic flexure	71 (4·5)
Transverse colon	149 (9·4)
Splenic flexure	54 (3·4)
Descending colon	37 (2·3)
Sigmoid colon	255 (16·1)
Rectosigmoid	74 (4·7)
Neoadjuvant therapy	
Yes	241 (15·2)
No	1344 (84·8)
Neoadjuvant type	
Short‐course radiotherapy	30 of 241 (12·4)
Long‐course CRT	211 of 241 (87·6)
Operative urgency	
Emergency	33 (2·1)
Urgent	85 (5·4)
Elective	1467 (92·6)
Differentiation	*n* = 1408
Undifferentiated	8 (0·6)
Poor	276 (19·6)
Moderate	1048 (74·4)
Well	76 (5·4)
pAJCC	
0 (pCR)	58 (3·7)
I	517 (32·6)
II	534 (33·7)
III	476 (30·0)
pT category	
0	62 (3·9)
1	302 (19·1)
2	313 (19·7)
3	784 (49·5)
4	124 (7·8)
Lymphovascular invasion	*n* = 1546
Yes	429 (27·7)
No	1117 (72·3)
Positive CRM	*n* = 1568
Yes	23 (1·5)
No	973 (62·1)
n.r.	572 (36·5)

* With percentages in parentheses unless indicated otherwise; values are

† mean(s.d.) and

‡ median (range).

CRT, chemoradiotherapy; pAJCC, pathological stage according to the AJCC; pCR, complete pathological response; CRM, circumferential resection margin; n.r., not recorded.

### Role of lymph node yield

In the univariable linear regression model several factors were associated with LNY (*Table* 2). LNY was lower in rectal cancer with increasing age and in patients undergoing long‐course (*versus* short‐course) neoadjuvant therapy, for open, hybrid and robotic surgical (*versus* laparoscopic) approaches, for undifferentiated cancers (*versus* well differentiated) and for lower rectal (*versus* upper) and left‐sided (*versus* right‐sided) tumours. Conversely, LNY was higher in urgent and emergency (*versus* elective) surgery, in hepatic flexure (*versus* caecal) tumours, in poorly differentiated (*versus* well differentiated) cancers, in stage I–III (*versus* stage 0) tumours, and in patients with mismatch repair protein deficiency. There was no significant association between LNY and sex, BMI or conversion of surgical entry.

**Table 2:** Univariable analysis of factors affecting lymph node yield

	β coefficient
Age (continuous)	−0·07 (−0·11, −0·03)
Sex	
M	1·00 (reference)
F	1·24 (0·98, 1·56)
BMI	0·07 (−0·15, 0·00)
Tumour location	
Colon	1·00 (reference)
Rectum	−2·16 (−2·98, −1·33)
Tumour site in colon	
Caecum	1·00 (reference)
Ascending colon	0·36 (−1·06, 1·77)
Hepatic flexure	3·42 (1·08, 5·76)
Transverse colon	0·26 (−1·50, 2·02)
Splenic flexure	0·37 (−3·20, 3·93)
Descending colon	−1·17 (−4·30, 1·96)
Sigmoid colon	−0·94 (−2·45, 0·56)
Neoadjuvant therapy	
No	1·00 (reference)
Yes	−3·22 (−4·32, −2·12)
Neoadjuvant therapy (rectal)	
No	1·00 (reference)
Yes	−2·33 (−3·63, −1·02)
Neoadjuvant therapy type	
Short‐course radiotherapy	1·00 (reference)
Long‐course CRT	−7·52 (−10·58, −4·45)
Surgical entry	
Laparoscopic	1·00 (reference)
Open	−1·19 (−2·19, −0·19)
Hybrid	−1·62 (−3·18, −0·06)
Conversion	−1·06 (−2·39, 0·27)
Robotic	−4·70 (−6·77, −2·63)
Operative urgency	
Elective	1·00 (reference)
Emergency	2·36 (−0·77, 5·49)
Urgent	4·47 (2·47, 6·48)
Rectal cancer location	
Upper	1·00 (reference)
Mid	−0·41 (1·64, 0·83)
Lower	−2·98 (−4·26, −1·71)
Differentiation	
Well	1·00 (reference)
Moderate	1·33 (−0·41, 3·08)
Poor	2·13 (0·18, 4·09)
Undifferentiated	−2·03 (−6·48, 2·41)
pAJCC	
0	1·00 (reference)
I	3·13 (1·07, 5·19)
II	5·50 (3·46, 7·55)
III	6·30 (4·18, 8·42)
dMMR IHC	
Normal	1·00 (reference)
MMR proteins absent	2·83 (0·88, 4·78)
AL procedure side	
Right	1·00 (reference)
Rectal	−2·53 (−3·47, −1·59)
Left	−2·45 (−3·34, −1·55)
Other*	5·27 (2·49, 8·05)

Values in parentheses are 95 per cent confidence intervals.

* Includes subtotal/total colectomy, Hartmann's procedure and proctocolectomy.

CRT, chemoradiotherapy; pAJCC, pathological stage according to the AJCC; dMMR, deficient mismatch repair protein; IHC, immunohistochemistry; MMR, mismatch repair; AL, anastomotic leak.

There were no significant differences between patients with low (below 12) or high (12 or above) LNY for a number of measured variables, including ASA grade (*P* = 0·978, χ^2^ test) and positive circumferential resection margin (*P* = 0·939, χ^2^ test). There were also no differences between surgeon (*P* = 0·104, χ^2^ test) or hospital site (*P* = 0·317, χ^2^ test). LNY was lower in patients with lymphovascular invasion (*P* = 0·017, χ^2^ test).

In multivariable analysis using linear regression, LNY below 12 was associated with increasing age, open surgery, robotic surgery (performed by 5 surgeons in 2012–2016) and elective surgery (*Table* 3). High LNY (12 or above) was associated with stage I–III *versus* stage 0 tumours. Patients with rectal cancer who had received neoadjuvant therapy showed a higher LNY for stage III pAJCC cancers alone and when in receipt of short‐course radiotherapy (*Table* 3).

**Table 3:** Multivariable analysis of factors affecting lymph node yield*

	β coefficient
Age (continuous)	−0·09 (−0·13, −0·05)
Rectal cancer	
No	1·00 (reference)
Yes	−1·01 (−2·03, 0·02)
Surgical entry	
Laparoscopic	1·00 (reference)
Open	−1·32 (−2·39, −0·24)
Hybrid	−1·04 (−2·62, 0·53)
Conversion	−0·90 (−2·27, 0·46)
Robotic	−3·74 (−5·90, −1·58)
Elective surgery	
No	1·00 (reference)
Yes	−3·44 (−5·14, −1·73)
pAJCC	
0	1·00 (reference)
I	2·44 (0·24, 4·63)
II	4·80 (2·59, 7·01)
III	5·33 (3·10, 7·57)
Rectal cancer with neoadjuvant therapy	
pAJCC	
0	1·00 (reference)
I	2·07 (−1·07, 5·21)
II	2·14 (−0·49, 4·77)
III	3·64 (0·88, 6·41)
Neoadjuvant therapy type	
Long‐course CRT	1·00 (reference)
Short‐course radiotherapy	7·03 (3·78, 10·27)

Values in parentheses are 95 per cent confidence intervals.

* Linear regression using lymph node yield (less than 12 or 12 or more) as a dichotomous variable.

pAJCC, pathological stage according to the AJCC; CRT, chemoradiotherapy.

Of the 1320 (83·9 per cent) of 1573 patients with colorectal cancer for whom follow‐up data were available, 130 (9·8 per cent) had disease progression within 5 years. Univariable survival analysis showed no statistical difference in DFS between low LNY (less than 12) and high LNY (12 or more) (*Table* 4). Multivariable analysis, adjusted for pathological stage, showed a lower risk of disease progression when 12 or more lymph nodes were collected (*Table* 4). A higher risk of progression was also evident for stage II or III compared with stage I tumours. No patient with stage 0 (pCR) progressed.

**Table 4:** Univariable and multivariable analysis of factors affecting disease‐free survival from colorectal cancer

	Hazard ratio
Univariable analysis	
LNY ≥ 12	0·80 (0·55, 1·17)
Multivariable analysis	
LNY	
< 12	1·00 (reference)
≥ 12	0·59 (0·41, 0·87)
pAJCC	
0	No disease progression
I	1·00 (reference)
II	3·89 (2·05, 7·37)
III	7·49 (4·05, 13·85)

Values in parentheses are 95 per cent confidence intervals. Predictor variables were included in analyses if significant at the 5 per cent level. LNY, lymph node yield; pAJCC, pathological stage according to the AJCC.

**Figure 1:**
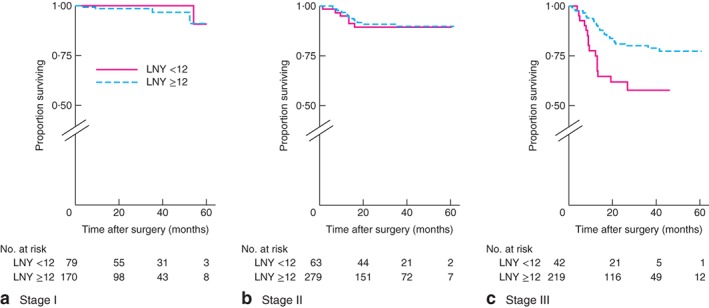
Kaplan–Meier analysis of disease‐free survival in patients with colonic cancer according to high (12 or more) and low (less than 12) lymph node yield (LNY): **a** stage I, **b** stage II, **c** stage III. **a** *P* = 0·355, **b** *P* = 0·751, **c** *P* = 0·002 (log rank test)

**Figure 2:**
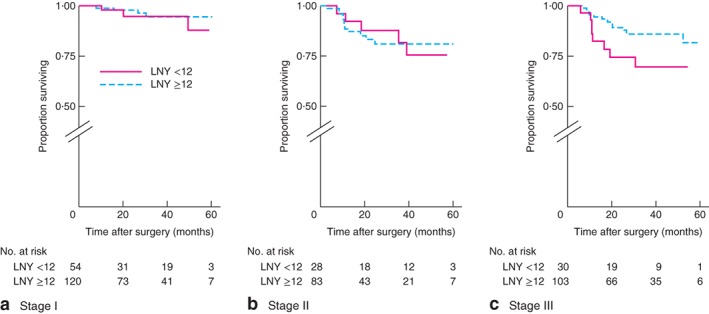
Kaplan–Meier analysis of disease‐free survival in patients with rectal cancer according to high (12 or more) and low (less than 12) lymph node yield (LNY): **a** stage I, **b** stage II, **c** stage III. **a** *P* = 0·493, **b** *P* = 0·912, **c** *P* = 0·058 (log rank test)

**Figure 3:**
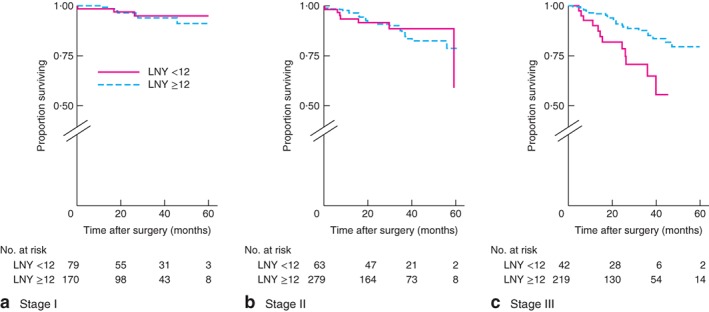
Kaplan–Meier analysis of overall survival in patients with colonic cancer according to high (12 or more) and low (less than 12) lymph node yield (LNY): **a** stage I, **b** stage II, **c** stage III. **a** *P* = 0·697, **b** *P* = 0·881, **c** *P* = 0·004 (log rank test)

**Figure 4:**
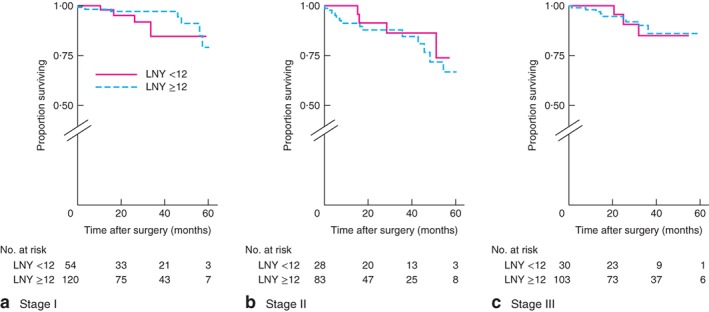
Kaplan–Meier analysis of overall survival in patients with rectal cancer according to high (12 or more) and low (less than 12) lymph node yield (LNY): **a** stage I, **b** stage II, **c** stage III. **a** *P* = 0·448, **b** *P* = 0·469, **c** *P* = 0·951 (log rank test)

After identifying an association between rectal cancer and low LNY (below 12), colonic and rectal cancer subdivisions were analysed further. DFS for colonic cancer was worse only for stage III tumours when data were stratified by low *versus* high LNY: stage I, *P* = 0·355; stage II, *P* = 0·751; stage III, *P* = 0·002 (log rank test) (*Fig*. 1). In contrast, there were no differences in DFS for rectal cancer when different stages were compared: stage 1, *P* = 0·493; stage II, *P* = 0·912; stage III, *P* = 0·058 (log rank test) (*Fig*. 2). OS for colonic cancer was worse only for stage III tumours when data were stratified by low LNY *versus* high LNY: stage I, *P* = 0·697; stage II, *P* = 0·881; stage III, *P* = 0·004 (log rank test) (*Fig*. 3). There were no differences in OS for rectal cancer when different stages were compared: stage I, *P* = 0·448; stage II, *P* = 0·469; stage III, *P* = 0·951 (log rank test) (*Fig*. 4).

When the 241 patients who had rectal cancer and underwent nCRT were examined, there was no association between LNY and DFS (hazard ratio (HR) 0·97, 95 per cent c.i. 0·91 to 1·03) or OS (HR 1·02, 0·97 to 1·07).

### Role of lymph node ratio

Higher LNR was associated with poorer DFS in multivariable analyses of colorectal cancer (*Table* 5). Risk of disease progression increased with increasing LNR quartile (0·25 and above).

**Table 5:** Multivariable analysis of the effect of lymph node ratio quartile on disease‐free survival in colorectal cancer

	Hazard ratio
LNRQ	
0 to < 0·0825	1·00 (reference)
0·0825 to < 0·25	1·94 (0·99, 3·81)
0·25 to < 0·5	2·59 (1·21, 5·53)
0·5–1	6·03 (2·85, 12·77)

Values in parentheses are 95 per cent confidence intervals. LNRQ, lymph node ratio quartile.

**Figure 5:**
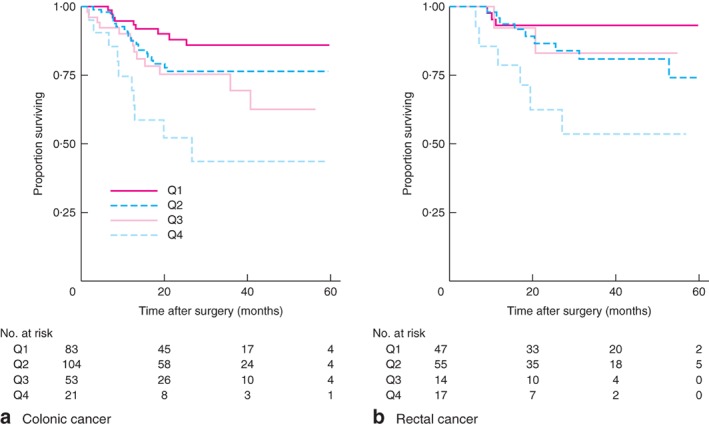
Kaplan–Meier analysis of disease‐free survival in patients with stage III **a** colonic and **b** rectal cancer according to lymph node ratio quartile: Q1, 0 to less than 0·0825; Q2, 0·0825 to less than 0·25; Q3, 0·25 to less than 0·5; Q4, 0·5–1. **a** *P* < 0·001, **b** *P* = 0·011 (log rank test)

When colonic and rectal cancer were considered separately, LNR was an independent predictor of DFS in stage III colonic cancer, with patients having poorer DFS with LNRs greater than 0·25 (*Table* 6). LNR of 0·5 or above was also a risk factor for patients with stage III rectal cancer (*Table* 6 and *Fig*. 5 *b*).

**Table 6:** Univariable analysis of the effect of lymph node ratio quartile on disease‐free survival in stage III cancers

	Hazard ratio
Colonic cancer stage III only	
LNRQ	
0 to < 0·0825	1·00 (reference)
0·0825 to < 0·25	1·86 (0·85, 4·06)
0·25 to < 0·5	2·64 (1·13, 6·17)
0·5–1	6·34 (2·56, 15·70)
Rectal cancer stage III only	
LNRQ	
0 to < 0·0825	1·00 (reference)
0·0825 to < 0·25	2·50 (0·67, 9·37)
0·25 to < 0·5	2·33 (0·39, 13·94)
0·5–1	7·20 (1·77, 29·37)

Values in parentheses are 95 per cent confidence intervals. LNRQ, lymph node ratio quartile.

In the cohort of 1320 patients with colorectal cancer for whom follow‐up data were available (83·9 per cent), 128 (9·7 per cent) died within 5 years of surgery. Although univariable analysis showed no difference in OS according to the number of lymph nodes harvested (HR 0·99, 95 per cent c.i. 0·97 to 1·02; *P* = 0·620) or with an LNY of 12 or more (HR 0·91, 0·62 to 1·34; *P* = 0·646), a higher LNR (as a continuous variable) was associated with lower OS (HR 6·99, 3·31 to 14·78). In multivariable analysis, poorer OS was associated with higher LNR quartile (*Table* 7).

**Table 7:** Multivariable analysis of the effect of lymph node ratio quartile on overall survival in colorectal cancer

	Hazard ratio
LNRQ	
0 to < 0·0825	1·00 (reference)
0·0825 to < 0·25	1·64 (1·03, 2·62)
0·25 to < 0·5	2·26 (1·22, 4·17)
0·5–1	4·59 (2·27, 9·28)

Values in parentheses are 95 per cent confidence intervals. LNRQ, lymph node ratio quartile.

**Figure 6:**
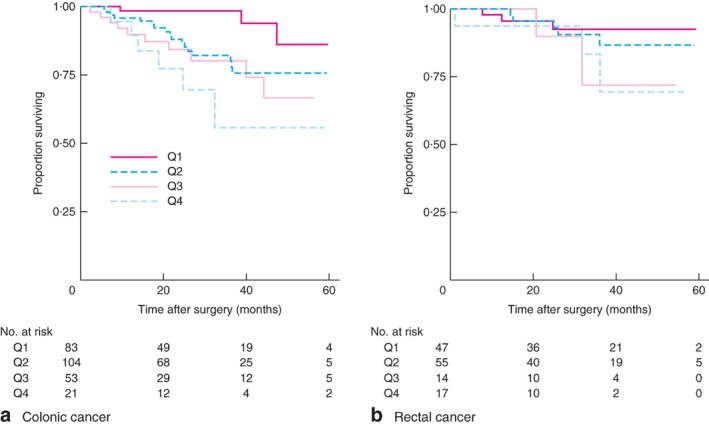
Kaplan–Meier analysis of overall survival in patients with stage III **a** colonic and **b** rectal cancer according to lymph node ratio quartile: Q1, 0 to less than 0·0825; Q2, 0·0825 to less than 0·25; Q3, 0·25 to less than 0·5; Q4, 0·5–1. **a** *P* = 0·007, **b** *P* = 0·391 (log rank test)

In the 241 patients with rectal cancer who had nCRT, poorer DFS was observed in patients with an LNR of 0·5–1 than in those with the lowest LNR (0–0·0825) (HR 11·24, 95 per cent c.i. 3·48 to 36·35). Compared with the reference group (LNR 0–0·0825), there was no association between LNR and OS in patients with an LNR of 0·0825 to less than 0·25 (HR 0·44, 0·06 to 3·36) or those with an LNR of 0·25 to less than 0·5 (HR 1·63, 0·21 to 12·45). When data were subdivided into colonic and rectal cancers, an LNR below 0·25 was associated with decreased OS in stage III colonic cancer (*P* = 0·007) (*Fig*. 6 *a* and *Table* 8). LNR was not a predictor of OS in stage III rectal cancer (*Table* 8 and *Fig*. 6 *b*).

**Table 8:** Univariable analysis of the effect of lymph node ratio quartile on overall survival in stage III cancers

	Hazard ratio
Colonic cancer stage III only	
LNRQ	
0 to < 0·0825	1·00 (reference)
0·0825 to < 0·25	4·44 (1·31, 15·08)
0·25 to < 0·5	5·90 (1·62, 21·49)
0·5–1	12·00 (2·92, 49·29)
Rectal cancer stage III only	
LNRQ	
0 to < 0·0825	1·00 (reference)
0·0825 to < 0·25	1·29 (0·30, 5·51)
0·25 to < 0·5	2·70 (0·45, 16·20)
0·5–1	2·78 (0·55, 14·09)

Values in parentheses are 95 per cent confidence intervals. LNRQ, lymph node ratio quartile.

## Discussion

LNY is an important prognostic factor in patients with stage II–III colorectal cancer, but evidence for stage I colorectal cancer is unclear^8^,^11^,^28^, ^29^, ^30^, ^31^. Although there are studies reporting differences in survival between low and high LNY in patients with Dukes' A/stage I colorectal cancer^10^,^15^, some indicate that LNY alone has no effect on survival outcome^32^, ^33^. The present study highlights that a substantial number of patients with stage I and II colorectal cancer have a low LNY. Nonetheless, both DFS and OS in stage I and II colonic and rectal cancers in the present cohort were excellent and not influenced by LNY. Low LNY in stage III colon cancer did, however, result in poorer DFS and OS. Low LNY had no effect on DFS or OS for rectal cancer.

LNR is generally considered more reliable as a prognostic tool in stage III colorectal cancer^5^,^16^,^23^,^34^. A systematic review and meta‐analysis^25^ involving 33 984 patients with stage III colorectal cancer described numerous different cut‐off LNR values. The authors concluded LNR was a more reliable prognostic factor than total number of positive nodes^25^. In the present study, a higher LNR resulted in worse DFS and OS in colorectal cancer. In addition, a high LNR correlated with a poorer DFS for both stage III colonic and rectal cancers. In stage III colonic cancer a high LNR also correlated with a poorer OS, whereas there was no correlation between LNR and OS in stage III rectal cancer. A high LNR in rectal cancer following neoadjuvant therapy resulted in worse DFS.

Neoadjuvant therapy is the current standard of care for locally advanced rectal cancer (T3–4 or node‐positive disease). A reduction in LNY secondary to radiotherapy is well recognized and the prognostic role of LNY in this setting is debatable. Miller and colleagues^18^ examined seven cohort studies and found a significant reduction in mean LNY in the nCRT group compared with that in patients who did not undergo nCRT (range 7–53 per cent). Despite some studies^35^, ^36^ showing that low LNY in rectal cancer treated with neoadjuvant therapy may be associated with poorer survival, there is emerging evidence^20^, ^21^,^37^ that low LNY in this clinical setting may not necessarily confer a poorer prognosis. A systematic review^38^ of 11 studies showed that although preoperative radiotherapy for locally advanced rectal cancer was associated with a lower LNY, most studies did not identify any adverse outcome in the low LNY group. This is consistent with the present findings in the subgroup of patients with locally advanced rectal cancer treated with nCRT.

In the present study, 23·5 per cent of all colorectal cancer resections were below the current benchmark of a minimum harvest of 12 lymph nodes; this is comparable to contemporary data from specialist centres^22^, ^39^, ^40^, but lower than that from other population‐based studies^6^, ^7^, ^41^. The majority of the variation in reported LNY is likely to be due to non‐modifiable patient‐specific factors, including tumour characteristics^6^. Factors such as older age, early tumour stage, type of operation (left‐sided/rectal operation compared with right‐sided) and use of neoadjuvant therapy in locally advanced rectal cancer have all been shown to be associated with low LNY^7^, ^28^, ^40^, ^42^, ^43^, ^44^. This was reflected in the present study, where lower LNY rates were associated with rectal cancer, elective surgery, stage 0 and I tumours, older patient age, left‐sided surgery and neoadjuvant treatment. Studies^43^, ^45^ have shown that operations performed by colorectal specialists in high‐volume centres are more likely to have a higher LNY than those performed by non‐specialists. It is possible that both surgeon and pathologist factors contributed to the LNY observed in the present study. In addition, fat clearance techniques can enhance lymph node assessment^46^, ^47^.

The present study has a number of limitations. Its observational nature is mitigated by the existence of a specific database for the collection of patient information. Although it is possible that there may have been differences in the way resection specimens were processed, the pathology departments followed the same AJCC guidelines. There was no statistical difference between the three hospitals in terms of LNY. A proportion of patients were lost to follow‐up – around 16·1 per cent over the study interval of 6 years. It is not known whether DFS and OS data would be different with full patient follow‐up (1573) in contrast to the 1320 patients for whom follow‐up data were available.

The principle of oncological resection is to create clear margins. Together with high ligation of the vascular pedicle, adequate lymph nodes can be harvested in the corresponding drainage basin. This allows accurate pathological staging of colorectal cancer that guides adjuvant chemotherapy. Nonetheless, despite following this principle, low LNY can be associated with earlier‐stage colorectal cancer as demonstrated in the present study. Low LNY in these clinical settings did not confer a poorer prognosis provided an adequate oncological resection and thorough examination of the surgical specimen had been performed.

These results support the use of LNR as a prognostic tool in stage III colorectal cancer to inform surveillance and treatment discussions (for instance with regard to adjuvant chemotherapy) following primary surgery. In addition, the use of LNR rather than LNY seems more logical in patients with rectal cancer treated with neoadjuvant therapy, given the tendency for a significant reduction in LNY.
